# Point-of-Care Electrochemical Diagnostic Developments for Multidrug-Resistant Bacteria: Role of Aptamers and Nanomaterials

**DOI:** 10.3390/bios16040186

**Published:** 2026-03-24

**Authors:** Kamna Ravi, Baljit Singh

**Affiliations:** 1 MiCRA Biodiagnostics Technology Gateway, Technological University Dublin (TU Dublin), D24 FKT9 Dublin, Ireland; 2 Health, Engineering & Materials Science (HEMS) Research Hub, Technological University Dublin (TU Dublin), D24 FKT9 Dublin, Ireland

**Keywords:** multidrug-resistant bacteria, antimicrobial resistance, point-of-care, lab-on-a-chip, nanomaterials, SELEX, electrochemical nano-aptasensor, antimicrobial susceptibility testing, microfluidics, 3D printing and smart connectivity

## Abstract

The unchecked and uncontrolled global spread of multidrug-resistant (MDR) bacteria is a serious challenge to healthcare and modern medicine, and demands diagnostic approaches that are rapid, sensitive, multiplexed, and reliable in point-of-care (POC) settings. At the interface of nanomaterials and aptamer-based biosensing, significant advances have been reported. The convergence of portable electrochemical sensing technologies, smartphone-based readout systems, and artificial intelligence (AI)- and machine learning (ML)-based data analysis is also playing a significant role in advancing this area. This perspective reflects on the most recent breakthroughs and translational developments in electrochemical nano-aptasensors for MDR bacterial detection, covering diagnostic targets and translation trends, nanomaterials advancements, aptamer engineering-integration, POC strategies and microfluidics platforms, and novel multimodal strategies that enhance accuracy, reliability, and efficiency in POC testing. Moreover, limitations and knowledge gaps in this area, as well as key considerations for sustainable development, large-scale manufacturing, and deployment of integrated electrochemical nano-aptasensors, are also highlighted. Electrochemical nano-aptasensors can pave the way for the transformation of MDR bacterial diagnosis, but need coordinated translational efforts for POC diagnostic advancements towards real-world applications.

## 1. Introduction

The rise in multidrug-resistant (MDR) bacteria and the emergence of antimicrobial resistance (AMR) constitute a persistent global health catastrophe. According to the latest World Health Organization (WHO) and Centers for Disease Control and Prevention (CDC) surveillance reports, AMR is estimated to take 10 million lives annually, with nearly half of these deaths (5 million) directly attributed to resistant infections [1,2]. At a molecular level, the emergence of MDR occurs through various pathways, including efflux pumps, enzymatic drug degradation, target site alterations, and reduced membrane permeability, all of which contribute to the reduced effectiveness of various antibiotics. Specific genetic markers of drug resistance, such as blaCTX-M, blaNDM, mecA, efflux pumps, etc., are used as targets both for diagnostic and therapeutic purposes, allowing for the early detection of resistance patterns and implementation of countermeasures [3,4,5]. The global healthcare crisis extends beyond mortality: prolonged hospitalizations, compromised efficacy of antibiotics, and exorbitant treatment costs threaten not only public health but also foundational medical care, including surgery and immunosuppressive therapy [6]. Early identification of pathogen(s) and resistance phenotypes during the course of infection is critical to effective containment, control, and treatment, but conventional culture and susceptibility testing require at least 24–72 h or longer, depending on the pathogen type, growth rate, and laboratory volume. The resulting diagnostic delay often necessitates empiric treatment with broad-spectrum antibiotics, thereby allowing resistance development and suboptimal clinical outcomes. The delay caused between infection and diagnosis remains a reason for failure in the treatment of sepsis, nosocomial outbreaks, and community-acquired MDR infections [7,8,9].

To address this gap, diagnostic solutions that provide rapid, reliable, and point-of-care (POC) results to inform infection control and antimicrobial stewardship measures remain a major priority. The emergence of POC diagnostic platforms that can rapidly identify both the pathogen and the corresponding resistance determinants could be considered a game-changer [10]. Recently, we have witnessed the accelerated pace of translational research that has finally started merging the challenge of connecting two highly promising technological paradigms. They include (i) nanomaterials, which represent engineered materials that are endowed with high conductibility, surface area, as well as optimal physicochemical properties; and (ii) aptamers, which are highly specific recognition molecules. Both of them improve the performance of electrochemical biosensors (EBs) and POC diagnostic systems for bacterial sensing.

Aptamers are uniquely synthesized, 3D-shaped oligonucleotides with high binding affinity and selectivity towards diverse target molecules. They are derived from the systematic evolution of ligands by exponential enrichment (SELEX) process and can serve as biorecognition modules for rapid label-free detection. Aptamers offer several advantages in biosensing applications, including high specificity and reproducibility, affinity for diverse targets, excellent chemical stability, and the ability to undergo conformational changes (binding-induced), which can collectively enhance sensor performance. They also add value to electrochemical diagnostics, in terms of improved overall stability, versatility, and batch-to-batch reproducibility, while allowing easy functionalization and regeneration [11,12]. Aptamers are of great interest as novel substitutes for antibodies in resource-limited and POC diagnostics.

These two components (nanomaterials and aptamers) are currently intersecting for a beneficial impact, as highlighted by recent reviews: nanomaterial-modified electrode interfaces based on gold nanoparticles (AuNPs), graphene, carbon nanotubes (CNTs), as well as metal–organic frameworks (MOFs) etc., are highly facilitating aptamer immobilization and signal transmission, whereas novel aptamers are propelling the detection boundaries for a number of bacterial genera as well as their resistance markers [13,14,15]. In the majority of electrochemical biosensing applications, these nanomaterials are deposited or assembled on popular and widely used electrodes, like glassy carbon electrodes (GCEs), gold electrodes, and screen-printed electrodes (SPEs), which are the basic building blocks of electrochemical analysis. GCEs are often employed in studies of electrochemical biosensing applications because of their remarkable chemical inertness, wide potential window, low background current, and reproducibility of surface properties, which make GCEs suitable for modification with nanomaterials and studying electron transfer processes [14]. Gold electrodes are of great interest for biosensing applications because they allow for the easy immobilization of thiolated aptamers using well-characterized Au-S bonds, which are widely employed for surface modification for biorecognition [15]. SPEs are gaining popularity for POC biosensing applications because of their low cost, disposability, and ease of mass production for portable sensing applications and biosensing configurations. The nanomaterials interface increases the electrode active area, facilitates efficient electron transfer rates, as well as signal amplification, hence increasing the overall sensitivity with improved performance and detection limits. Additionally, the modification of the electrode surfaces using nanomaterials offers new functional groups that improve the density of aptamer loading and target accessibility, which can improve analytical performance [15]. Furthermore, lab-on-a-chip microfluidics, smartphone detection capabilities, as well as artificial intelligence (AI) and machine learning (ML) processing for data interpretation, are currently introducing such techniques to transform these platforms into deployable tools [16].

While pre-existing reviews have examined individual advancements in aptamer technologies and electrochemical biosensing [14,15,16,17,18,19,20,21,22,23,24,25], they are typically in a broad context rather than being specifically centered on the development of electrochemical nano-aptasensors within POC diagnostic platforms for MDR bacterial detection. For instance, the review by Zhou et al. discusses the systematic evolution of electrochemical sensors and highlights the role of nanomaterials and different biorecognition elements, enabling miniaturized diagnostic systems for bacterial detection; however, advances in AI/ML data collation and their integration remain largely underexplored [14]. Similarly, Khan et al. focus on electrochemical approaches for detecting antibiotic-resistant bacteria, emphasizing developments in electrode materials, bio-receptor strategies, and microfluidic integration for antimicrobial susceptibility testing [16]. Fatah et al. highlight the advantages of combining aptamers with metal–organic frameworks to enhance signal amplification and detection sensitivity in pathogen sensing platforms [17]. Additionally, electrochemical aptasensors have often been discussed in the context of their application in detecting foodborne bacteria [18,22] and various bacterial by-products [19,20,21,22,23,24,25], with very limited attention to their integration and development with nanomaterials and AI/ML strategies into POC diagnostic platforms.

However, these reviews primarily discuss biosensing materials, sensing mechanisms, or individual recognition strategies separately and do not specifically address the integrated development of electrochemical nano-aptasensors within POC diagnostic frameworks for MDR bacterial detection. In contrast, this perspective provides a focused overview of the convergence of nanomaterial-enabled electrochemical aptasensing, aptamer engineering and integration, and POC diagnostic platforms for rapid MDR bacterial detection. Specifically, recent developments in nanomaterial-based electrochemical aptasensors, aptamer engineering, and their integration with microfluidic lab-on-a-chip technologies have been highlighted. Additionally, an emphasis has been placed on the emerging AI/ML-assisted analytical tools and their importance in outlining a translational pathway toward next-generation POC diagnostics. Furthermore, the limitations, knowledge gaps, and key considerations for scalable fabrication, system integration, and real-world deployment of electrochemical nano-aptasensors are brought to the foreground. By emphasizing the synergistic interaction between nanotechnology and aptamer-based molecular recognition, this perspective article outlines how electrochemical nano-aptasensor POC platforms are advancing toward rapid, sensitive, and clinically deployable detection of MDR bacteria, thereby underscoring the need for such advancements.

## 2. Recent Developments

The past few years have witnessed a shift from proof-of-concept biosensors towards clinically translatable platforms for the direct detection of MDR bacteria in patient-derived specimens. Various literature and reports describe a heightened emphasis on real-world matrices like blood, urine, wound exudate, respiratory secretions, and stool that better mimic the complex environments where immediate MDR diagnosis is critical [14]. Recent developments have focused on integrating multiplex detection, resistance profiling, and phenotypic susceptibility testing in singular, self-contained units with the ability to operate under POC scenarios. It is worth mentioning that various materials and nanomaterials were used as electrodes and electrode modifiers in electrochemical biosensing, such as gold nanoparticles (AuNPs), graphene (C), carbon nanotubes (CNTs), metal–organic frameworks, and conductive polymers (CPs). These materials have been studied and reported previously for the construction of electrochemical sensors for small molecules, including pharmaceutical compounds and metabolites. These materials are known to exhibit good electrical conductivity, as well as the ability to be functionally modified and exhibit good biocompatibility, which are essential requirements in the context of bacterial diagnostics, where efficient signal transduction of biological probes, such as aptamers, antibodies, and nucleic acids, is a critical need [7]. These materials will help in advancing miniaturized POC systems for bacterial and AMR detection.

Moreover, the current state-of-the-art of electrochemical biosensors is different from the earlier generation of specific analyte sensors, as the focus has now shifted from individual sensing electrodes to the development of integrated and multiplexed diagnostic systems. This is due to recent developments in disposable SPEs, microfluidic sample management systems, portable potentiostats, and smartphone-based data acquisition systems having enabled the direct translation of the existing electrode chemistries into miniaturized and scalable diagnostic systems [17].

### 2.1. Diagnostic Targets and Translation Trends in EBs

Recent studies have demonstrated the potential of EBs to detect MDR pathogens such as *Escherichia coli* (*E. coli*), *Klebsiella pneumoniae* (*K. pneumoniae*), *Pseudomonas aeruginosa* (*P. aeruginosa*), *Acinetobacter baumannii* (*A. baumannii*), and *Staphylococcus aureus* (*S. aureus*) directly from samples without the need for laboratory-based culture and analysis processes that are time-consuming and expensive. These systems detect either species-specific RNA or DNA sequences, resistance genes (such as blaCTX-M, blaNDM, mecA, tetM), or resistance proteins (antibodies, efflux pumps, and enzymes) and metabolic fingerprints. An additional shift is marked by the introduction of multiplexed detection panels that facilitate the identification of multiple pathogens. This is particularly helpful in polymicrobial infection or healthcare-associated infections (HAIs), where empirical therapy has a tendency to fail. A significant enhancement of the sensitivity, specificity, and portability of such biosensing platforms has also been observed. For instance, Qazi et al. (2024) reviewed various EBs with advanced diagnostic targets (including antibodies, antigens, LPS, proteins, enzymes, and nucleic acids) available for *E. coli* detection [26]. They were divided into two categories based on their biological recognition process, i.e., biocatalytic devices and affinity sensors. While biocatalytic devices involve enzymes, tissues, or whole cells, the affinity sensors make use of the specific binding interaction between a biomolecule and the analyte. They were reported to exhibit improved sensitivity based on nanomaterial-modified electrodes, suggesting rapid and label-free detection with LODs within the range of 1–112 CFU/mL [26].

**Figure 1:**
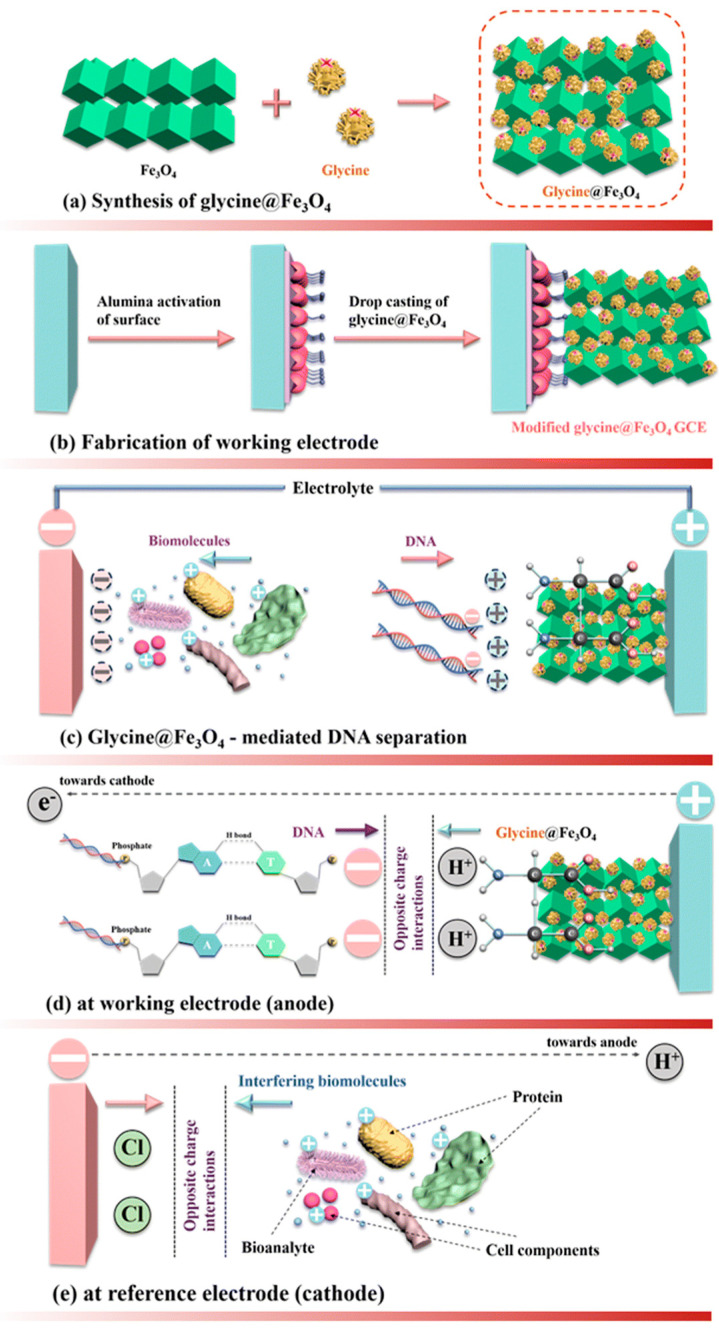
Schematic of the DNA biosensor platform using glycine@Fe_3_O_4_. (**a**) Synthesis of glycine@Fe_3_O_4_ nanoparticles, (**b**) fabrication of the working glassy carbon electrode using glycine@Fe_3_O_4_, (**c**) glycine@Fe_3_O_4_-mediated DNA sensing, (**d**) DNA attracted towards the anode during DNA sensing through opposite charge attraction, and (**e**) interfering biomolecules attracted towards the cathode through opposite charge attraction. Reproduced from Ref. [27] (Journal of Materials Chemistry B, RSC), copyright (2025) with permission from The Royal Society of Chemistry (RSC). This article Ref. [27] is licensed under a Creative Commons Attribution-NonCommercial 3.0 Unported License.

Similarly, Gambhir et al. (2025) reported a glycine-functionalized iron oxide nanoparticle-based electrochemical biosensor (glycine@Fe_3_O_4_-mediated DNA biosensor platform, Figure 1) possessing the potential for the identification of *K. pneumoniae* DNA (specifically antimicrobial resistance genes associated with *K. pneumoniae*: SHV, TEM, CTX-M, OXA-1) with good selectivity and stability even in complex biological samples with LODs reported as 3.27 nM (linear range: 30–90 nM) and 3.94 nM (linear range: 120–270 nM) [27]. For *P. aeruginosa*, Zhang et al. (2024) reported a dual-mode biosensor that incorporates HCR, CRISPR-Cas12a, and a DNA tetrahedron (Td) containing methylene blue (MB) based on electrochemical and colorimetric readouts with a multiple-signal amplification strategy that displays a LOD of 14 CFU/mL [28].

Further advances include bacteriophage-enabled recognition systems, such as a nanoflower-based electrochemical biosensor hybrid, which allows for the direct detection of viable *A. baumannii* cells (whole cells) in real-world matrices (serum and plasma) with a LOD up to 3 CFU/mL, as described by Wang et al. (2025) [29]. In another example, Guo et al. (2024) reported the use of an RPA-CRISPR/Cas12a-based biosensor with a conductive nanocomposite for ultrasensitive detection of *S. aureus* with a LOD of 3 CFU/mL, combining the benefits of genetic-level functions with high throughput [30].

In addition, Kao and Alocilja (2025) noted the heightened participation of biosensors in sensing carbapenem resistance genes with a LOD of 10^5^ CFU/mL, highlighting their possible integration into AMR surveillance systems [31]. Collectively, these developments represent a progressive movement towards miniaturized and multiplexed electrochemical biosensor technologies with the potential to transform clinical diagnostics and infection control measures [31].

### 2.2. Nanomaterials-Driven EBs

**Figure 2:**
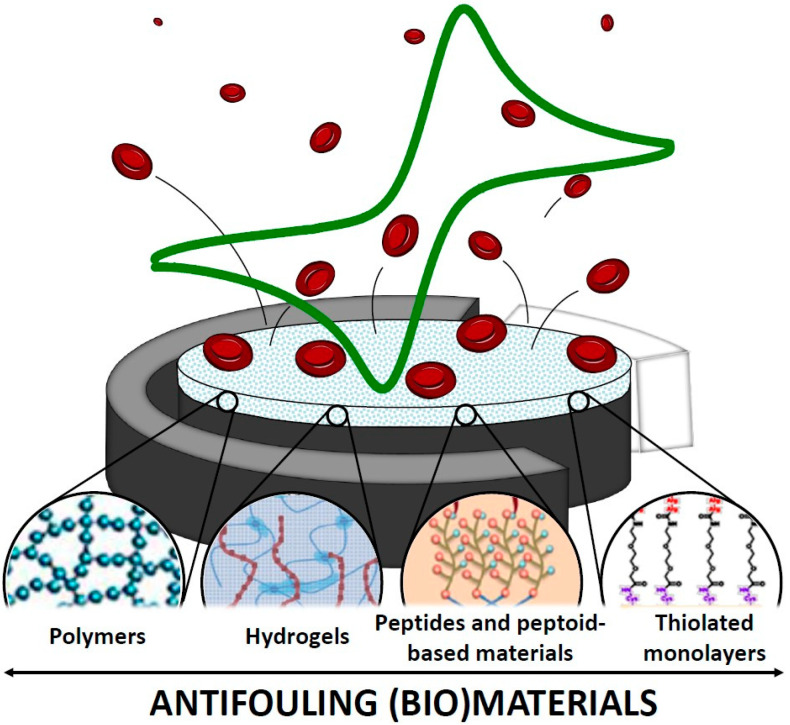
Antifouling (bio)materials for electrochemical (bio)sensing. Reprinted from Ref. [34] (International Journal of Molecular Sciences, MDPI) © 2019 by the authors. Licensee: MDPI, Basel, Switzerland. This article Ref. [34] is an open-access article distributed under the terms and conditions of the Creative Commons Attribution (CC BY) license.

Nanomaterials form the basis for EBs translation. Their availability has moved from theoretical optimization to direct implementation of diagnostic capacity in human specimens: gold nanoparticles (AuNPs) and graphene nanocomposites enabled enhanced electron transfer and aptamer immobilization for the detection of MDR *E. coli* and *K. pneumoniae* in urine and sputum specimens, with maintained specificity at high sample protein content, reported LODs of 0.5 × 10^−7^ ng/μL and 1 × 10^−7^ ng/μL, respectively [32]. CNT scaffolds and MOFs were employed in capturing and concentrating bacterial cells from blood, reported to enhance assay sensitivity for *A. baumannii* and methicillin-resistant *S. aureus* (MRSA) [33]. Conductive polymer composites coated with antifouling (bio)materials are reported to exhibit long-term signal stability on samples, suggesting overcoming a major drawback of matrix interference (Figure 2) [34]. These nanostructured interfaces not only decrease detection or analysis time but also increase reproducibility and portability, allowing miniaturized battery-operated or smartphone-linked POC devices to be applied in emergency rooms, intensive care units (ICUs), and resource-limited clinics.

### 2.3. Aptamer Engineering and Integration (EBs)

Recent advancements in SELEX have generated aptamers with greater specificity under the complexity of biological conditions. However, some challenges are still associated with the generation of aptamers with optimal binding properties even in the context of complex bacterial biofilms or heterogeneous clinical samples. These include off-target binding, susceptibility to nuclease degradation, and instability of aptamer conformations under different temperature and/or pH conditions. Current advances, including post-SELEX chemical modification, PEGylation, backbone stabilization, and the application of microfluidic SELEX technologies, are being used to circumvent some of these issues. In addition, rational aptamer design, including the application of computational modeling and molecular dynamics simulations, has been used to identify aptamers with optimal binding properties to well-characterized bacterial targets, potentially revealing novel targets for diagnostics as well as therapy [35,36]. In Wong et al. (2025), the authors trace the evolution of aptamer selection from classical cell-SELEX to tissue-SELEX modalities like morph-X-SELEX, ex vivo SELEX, and microfluidic tissue platforms [35]. These approaches were reported to improve aptamer recognition of native tissue targets and enhance drug delivery (especially for ocular, pulmonary, cardiac, and tumor tissues) [35]. Importantly, these next-generation methodologies can be extremely beneficial for bacterial diagnostics by placing an emphasis on selective and efficient aptamer selection that recognizes pathogens in complex but physiologically relevant settings, thereby enhancing the specificity and overall performance of infection detection.

**Figure 3:**
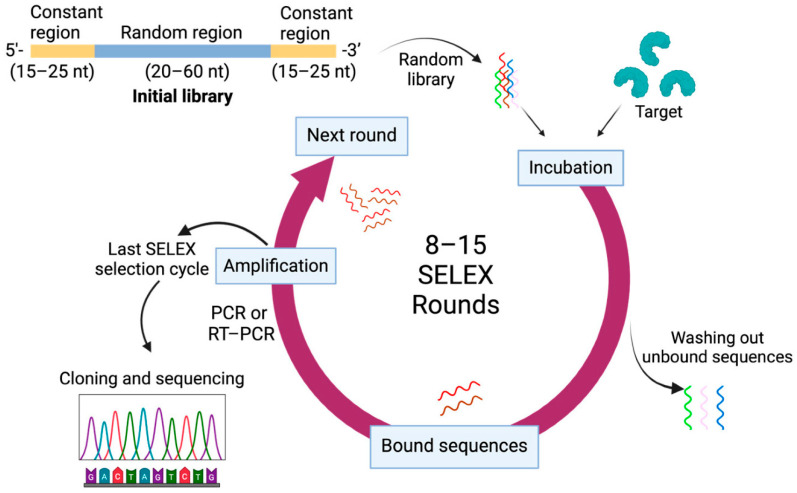

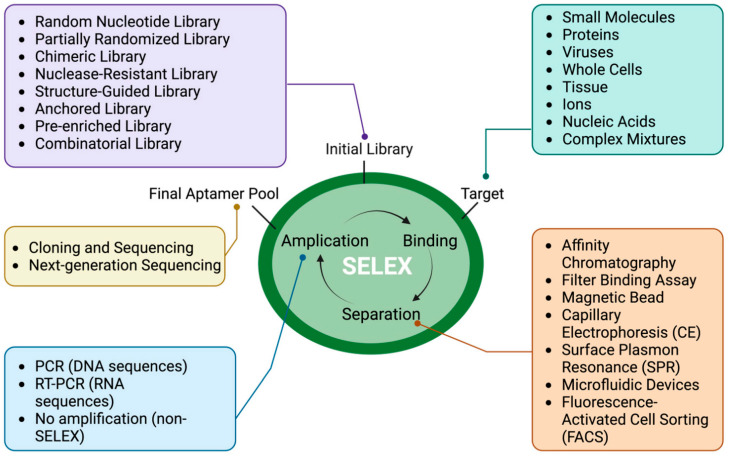
**Top**: Process of systematic evolution of ligands by exponential enrichment (SELEX). **Bottom**: Overview of common SELEX variations. Reprinted from Ref. [39] (Pharmaceutics, MDPI) © 2024 by the authors. Licensee: MDPI, Basel, Switzerland. This article Ref. [39] is an open-access article distributed under the terms and conditions of the Creative Commons Attribution (CC BY) license.

In Didarian et al. (2025), a discussion of the advancements in SELEX approaches (namely microfluidic SELEX, capillary electrophoresis SELEX, in vivo SELEX, high-throughput sequencing, and post-SELEX chemical modification) for aptamer stability, off-target binding repression, nuclease degradation prevention, and acceleration of selection has been established [36]. They also highlight that improved platforms have pushed aptamer sensitivity into more demanding detection regimes. Concurrently, Legen and Mayer (2025) report a robot-assisted, automated capture SELEX process to make RNA aptamers bind small molecules using high throughput and reproducibility [37]. Chen et al. (2025) describe screening for an aptamer against the human CD20 extracellular protein (expressed in *E. coli*), using iterative SELEX cycles and binding assays; the authors identify binding affinities (K_d_) of the aptamers selected [38]. Despite their high potential in diagnostics, there are currently only a few established aptamers that have been utilized primarily for the detection of non-infectious diseases like macular degeneration. However, with continued innovation, tremendous promise lies with aptamers in further extending their use in bacterial diagnostics (Figure 3) [39].

Aptamer-based electrochemical sensors are well known to be effective for MDR pathogen and resistance determinants detection due to their high selectivity. Aptamers have some advantages over antibodies, such as chemical stability, easier synthesis and modification, reduced batch-to-batch variability, and the capacity to withstand harsh environmental conditions without compromising their activities. These advantages make aptamers suitable candidates for incorporation into electrochemical biosensors and POC devices. Although antibody-based biosensors have found broad applications in commercial devices due to the extensive history and validation of these molecules, aptamers have the potential to replace them as the next generation of biosensors due to the capacity to generate these molecules in vitro using SELEX, which can be used to generate aptamers against new targets and scale up production at low costs [36,37,38]. Throughout recent years, some significant applications have been reported. For instance, an electrochemical aptasensor with vancomycin-modified screen-printed carbon electrode (SPCE) was reported to detect *S. aureus* and *B. cereus* with a detection limit of 100 CFU/mL in less than 45 min [40]. A sandwich-type electrochemical aptasensor with the SPCE modified with MIL-101(Cr)/multi-walled carbon nanotubes (MWCNTs) based on a signal amplification strategy was developed to detect *P. aeruginosa* with a LOD of 1 CFU/mL (σ = 3) [41].

In another study, a novel electrochemical aptasensor was reported to detect *E. coli* O157:H7 using a GCE modified with a reduced graphene oxide-poly(vinyl alcohol) and gold nanoparticles nanocomposite (AuNPs/rGO–PVA/GCE). The linear relationship of the *E. coli* concentration and the peak current in the range from 9.2 CFU mL^−1^ to 9.2 × 10^8^ CFU mL^−1^ was reported in the study with a LOD of 9.34 CFU mL^−1^ [42]. Another electrochemical aptasensor study incorporates a cDNA–ferrocene–zeolitic imidazolate framework-8 (cDNA-ZIF-8-Fc) signal tag for *S. aureus* detection and reported a linear concentration range of 15.0–1.5 × 10^7^ CFU mL^−1^, with a LOD of 5.3 CFU mL^−1^ [43]. Additionally, Chen et al. reported arrays of aptasensors and multiplexed aptasensors, which monitor various MDR bacteria in POC settings, which distinguish between resistant and susceptible isolates based on distinct electrochemical signatures [44]. Though currently dominated by antibody-based biosensors in commercial devices because of established regulatory acceptance and validation, aptamer-based biosensors and systems continue to attract interest in research and development due to their potential for design flexibility, stability, and compatibility with nanostructured materials for signal transduction mechanisms.

### 2.4. POC Platforms, Mass Production, and Smart Data-Connectivity

**Figure 4:**
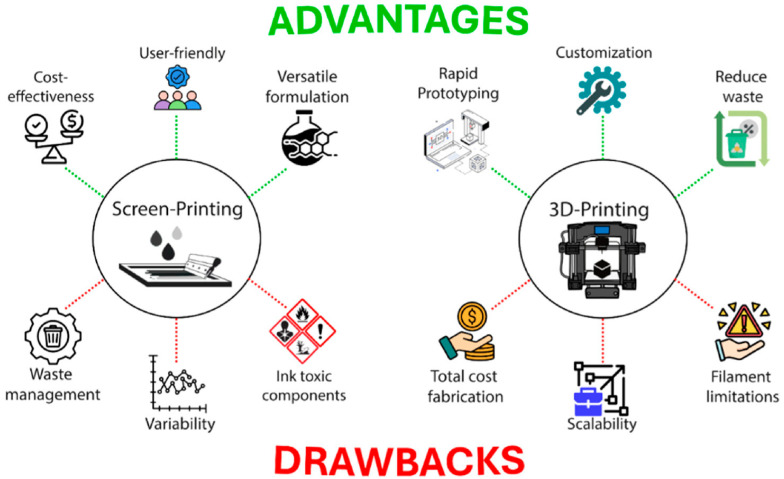
Advantages and drawbacks of the screen- and 3D-printing technologies. Reprinted from Ref. [46] (ECS Sensors Plus, The Electrochemical Society), copyright (2025). This is an open-access article Ref. [46] distributed under the terms of the Creative Commons Attribution 4.0 license (CC BY).

Emerging advances in translational science have focused on incorporating sophisticated chemistries within small and user-friendly diagnostic devices. Microfluidic lab-on-a-chip devices now combine multiple analytical processes (i.e., filtration of the sample, bacterial lysis, isolation of nucleic acids, amplification, and electrochemical detection) into a single small cartridge. These integrated systems have already been assessed with real-life matrices like urine and sputum for the concurrent detection of MDR *K. pneumoniae* and *P. aeruginosa* [45]. For enhanced accessibility and scalability, screen-printed electrodes (SPEs) on inexpensive paper or plastic flexible polymer substrates are increasingly used with disposable and mass-producible diagnostic forms that are suited for use in POC testing. SPEs combined with Bluetooth potentiostats and smartphone-based interfaces provide real-time, wireless data transfer to cloud servers for rapid result interpretation, epidemiologic mapping, and telemedicine consultation. In recent times, by exploiting the rapid prototyping ability of 3D-printing technology, the production of intricate designs and the integration of multiple functionalities in a singular device were made possible [46]. These complex configurations improve the overall sensor performance while significantly reducing waste and manufacturing time, thereby expanding the possibilities for innovative sensor development. However, many issues regarding materials availability for 3D printing persist. For instance, only a few printable materials possess adequate electrical, mechanical, and chemical properties for the fabrication of advanced sensors. In addition, a number of advanced 3D printing techniques used for sensor fabrication, such as multi-material or micro-resolution 3D printing, require specialized equipment or conditions for 3D printing, which are still not available on a large scale. However, these issues are being addressed by developing new printable materials with adequate properties for sensor fabrication, increasing resolution, and making 3D printing more affordable; the requirement for specialized equipment is currently also being addressed (Figure 4) [46].

A particularly compelling advancement is the hybrid phenotypic–electrochemical assay, where impedance is assessed after short exposure to antibiotics (1–2 h) to forecast bacterial susceptibility. Such developments directly answer the clinical query of resistance or susceptibility of an isolate, greatly reducing the time taken for treatment of life-threatening infections [47]. These algorithmically designed sensing platforms are increasingly being integrated into Internet of Things (IoT) infrastructure, enabling real-time data exchange between decentralized POC diagnostics, electronic health records (EHRs), and public health databases. IoT-backed AMR dashboards integrate spatiotemporal ML models to pattern resistance spread in healthcare networks, while AI-backed mobile diagnostic hubs advance these capabilities to resource-limited environments through edge-computing infrastructure. Concurrently, scalable microfabrication and roll-to-roll nano-printing technologies enable high-throughput fabrication of lab-on-a-chip cartridges and sub-micron resolution disposable electrode arrays, enabling cost-effective deployment of globally networked, intelligent diagnostic platforms for AMR detection and control [48].

The incorporation of Internet of Medical Things (IoMT) networks enables automated collection from dispersed diagnostics units, which facilitates AMR surveillance on a bigger scale and predictive outbreak modeling. Recent research shows how the interface between AI, ML, and nanoscale biosensing is facilitating a new wave of adaptive diagnostic platforms for AMR detection. Yammouri and Lahcen (2024) highlight the way wearable sensors with AI support and smart POC devices combine self-learning algorithms with multimodal transduction interfaces in EBs to enable continuous, personalized detection of infection biomarkers [49]. Nashruddin et al. (2024) also highlight the movement of big data and big health technologies from theory to practice and describe the recent advancements in the development of AI-powered electrochemical biosensors and their applications in real-time disease detection and personalized healthcare [50]. Pious et al. (2025) proceed to outline nanoscale sensing approaches (graphene-based field-effect transistors (GFETs)) that can detect antibiotics like gatifloxacin at the femtomolar level and enable unprecedented analytical sensitivity in AMR monitoring [51].

### 2.5. Novel Multimodal POC Strategies

**Figure 5:**
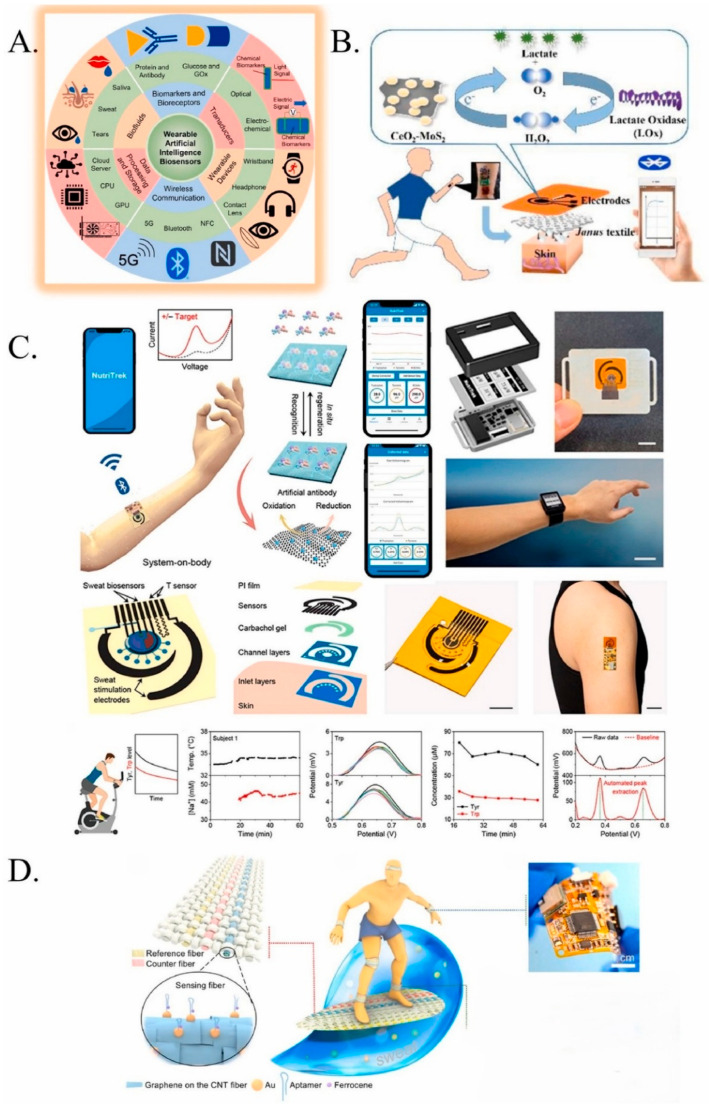
(**A**) Functional components of AI-assisted wearable biosensing systems. (**B**) Nanozyme–enzyme electrochemical biosensor for sweat lactate monitoring. (**C**) The ‘NutriTrek’ wearable biosensor for metabolic monitoring through sweat biosensing as a mobile application and a smartwatch and wearable system evaluation with custom voltammogram analysis using real-time calibrations. (**D**) A wearable electrochemical fabric for cytokine monitoring. Reprinted from Ref. [50] (Heliyon, Elsevier), copyright (2024). This is an open-access article Ref. [50] distributed under the terms of the Creative Commons Attribution 4.0 license (CC BY).

Sophisticated research integrates electrochemical aptasensors with molecular and computational strategies, improving accuracy: CRISPR/Cas-based signal amplification mechanisms together with electrochemical transducers have detected resistance genes blaNDM-1 and mcr-1, outpacing the majority of PCR-based systems in terms of speed and sensitivity [52]. AI and ML integration make the sensor system adept at the identification, categorization, characterization, and projection of various data patterns. AI and ML algorithms operate on large electrochemical data sets to sort resistance profiles and deconvolute multiplex signal noise, a process that enhances diagnostic specificity in patient samples [53]. Nanopore sequencing is also combined in some cases downstream from rapid POC electrochemical screening for confirmatory resistance gene analysis, providing an overall two-tier diagnostic system. In the transition from theory to practice, electrochemical sensors powered by AI, ML, and IoMT offer significant opportunities for the development of POC disease detection and personalized healthcare interventions (Figure 5) [50].

These multimodal systems represent a paradigm shift towards networked, smart diagnostics that are capable of detecting, validating, and reporting MDR infections at record speed [46,54]. From such methods, recent proof-of-concept demonstrations have been made of integrated microfluidic platforms that combine electrochemical sensors with optical and fluorescence-based detection in one device [55]. These systems enable detection of multiple resistance genes, metabolic biomarkers, and host immune markers within 60 min [56]. By overlaying complementary detection modalities, researchers can cross-validate internally for signals and reduce false positives while enhancing the validity of rapid, POC MDR screening. In addition, AI-driven pattern recognition software can combine the multimodal readouts to generate an integrated resistance profile, effectively producing a “digital twin” of the pathogen resistance topology [57].

**Figure 6:**
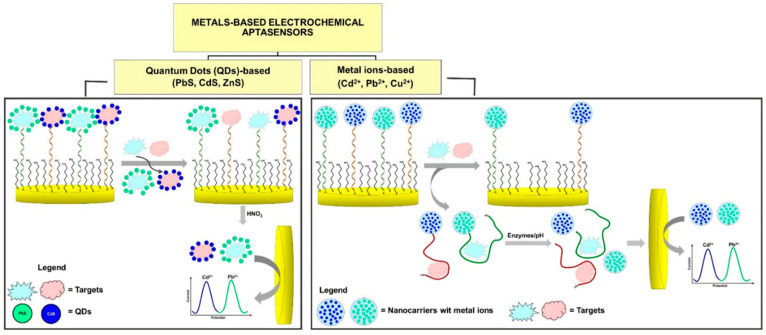
General sensing mechanisms of multiplex electrochemical aptasensors (MEAS) based on quantum dots, metal chalcogenide NPs, and heavy metal ions. Reprinted from Ref. [61] (Sensors, MDPI), © 2021 by the authors. Licensee: MDPI, Basel, Switzerland. This article Ref. [61] is an open-access article distributed under the terms and conditions of the Creative Commons Attribution (CC BY) license (https://creativecommons.org/licenses/by/4.0/).

Other recent strategies of synergistic combinations of nanoparticle-amplified spectroscopy, surface plasmon resonance (SPR), and microelectromechanical systems (MEMS) enhance MDR identification. For example, functionalized AuNPs can amplify electrochemical and optical signals from resistance determinants [58], whereas MEMS-based platforms allow highly sensitive real-time measurement of bacterial growth and drug response at the microscale [59]. Combining these technologies with cloud-based analytics offers remote interpretation and early warning of resistant outbreaks and reconfigures MDR diagnostics into predictive, rather than purely reactive, frameworks. These advanced multimodal solutions paint the picture of the future of POC platforms, where rapid detection, multistate validation, and networked data intelligence converge to accelerate therapeutic decision-making [60]. Additionally, Grabowska et al. reviewed and reported some current approaches employed in the development of multiplex electrochemical aptasensors with high specificity and affinity for the detection of multiple analytes through a singular analysis [61]. They group these methods into three sub-categories which include: (1) organic redox probes (anthraquinone, ferrocene, hemin groups, methylene blue or thionine) attached to aptamers which can alter certain signals or properties; (2) PbS, CdS, ZnS quantum dots, or Pb^2+^/Cd^2+^ metals/ions, which give label-specific electrochemical signals; (3) enzyme label with nanomaterials to give amplified electrochemical signals via catalytic reactions (Figure 6) [61].

## 3. Limitations, Knowledge Gaps, and Considerations

### 3.1. Limitations

Despite significant progress in POC electrochemical diagnostics for MDR bacteria, several limitations have been observed:

#### 3.1.1. Biological Complexity of Clinical Samples

Clinical samples like blood, urine, sputum, and wound exudates contain high levels of interfering substances like proteins (albumin and globulins), electrolytes and salts, whole cells, cellular debris, lipids, and small-molecule metabolites. In EBs, they can greatly impact overall performance by inducing nonspecific adsorption of samples, blocking active binding sites, fouling electrodes, impeding electron transfer, or generating background currents that mask analytical signals. Moreover, changes in pH, ionic strength, and viscosity can also complicate target recognition and signal transduction [62]. Consequently, while many aptamer–nanomaterial-based sensing platforms have been shown to be sensitive and specific in controlled buffer solutions, their analytical performance can be severely compromised in real clinical samples because of decreased binding efficiency, increased noise, reduced signal-to-noise ratio, and overall lack of reproducibility and reliability [63,64]. Although promising sensitivity and selectivity are demonstrated in pilot studies, performance may be compromised in complex clinical samples such as blood, urine, or wound exudates, owing to biofouling, non-specific adsorption, and molecular interferences, which affect the reliability of the signals, especially in multiplexed formats or fast phenotypic assays. Antifouling surfaces, surface chemistry optimization, as well as sample pretreatment, are some of the strategies employed to improve performance; nevertheless, comprehensive validation of such samples remains a challenge, as rigorous clinical studies are required [65].

#### 3.1.2. Aptamer Stability in POC Settings

The correct three-dimensional conformation of the nucleic acid sequence of aptamers must be maintained throughout in order to bind the target with the appropriate affinity and specificity. In POC applications, which can be heavily decentralized, elevated temperatures, extreme pH values, and repeated freeze–thaw cycles may induce the denaturation of the nucleic acid sequence. Moreover, the concentration of the buffer and the presence of divalent ions, which are very important in maintaining the structure of the nucleic acid sequence, may also be less controlled in such environments. Additionally, the nucleic acid sequence can be easily degraded by the various nucleases prevalently available in biological fluids like blood, urine, sputum, etc. [65].

#### 3.1.3. Reproducibility of Nanomaterial Synthesis

Nanomaterials like AuNPs, graphene derivatives, CNTs, and MOFs are characterized by a high degree of batch-to-batch variability due to heightened sensitivity to small changes in synthesis time and overall conditions, reagent purity, and temperature. Changes in the overall properties of the nanomaterials used may have an effect on the electron transfer rates, catalytic activity, and adsorption properties. In addition, small differences in the oxidation state or defect concentration in graphene derivatives may cause a dramatic difference in conductivity. In a similar vein, small differences in the alignment state, length, or purity of CNTs may also be contributing factors. Finally, small differences in the coordination state or pore size in MOFs may play a role in the determination of the binding affinities. These differences in properties may cause a dramatic difference in the sensor signal, sensitivity, selectivity, and stability [66,67].

#### 3.1.4. Multiplexing and Sensitivity-Preparation Trade-Offs

Multiple electrochemical diagnostic platforms emphasize the use of highly specific recognition elements like antibodies, aptamers, or nucleic acid probes that specifically target one organism at a time. Though this strategy can provide excellent sensitivity and specificity, it fails to address infections that are typically polymicrobial. Furthermore, AMR profiling not only requires the identification of the causative microorganism but also the simultaneous detection of specific resistance determinants, such as genes that code for β-lactamases, carbapenemases, or efflux pumps, which can be transferred horizontally among different microorganisms [68].

Moreover, ultra-low detection limits in diagnostic assays can also be achieved by using signal amplification methods, and pre-enrichment methods are used to increase the analyte concentration before the assay. The increase in time taken by the assay due to these methods can not only delay decision-making but also increase the risk of contamination and user error. Therefore, although signal amplification and pre-enrichment methods can be very useful in achieving low LODs in diagnostic platforms, they can also compromise the simplicity, speed, and portability of the sensor necessary for effective POC testing [69].

#### 3.1.5. Regulatory and Clinical Translation Barriers

A low number of aptamer–nanomaterial-based electrochemical biosensors targeting MDR bacteria have moved from laboratory validation to comprehensive clinical evaluation, despite promising results in preclinical trials. Their validation in large multicentre clinical trials is necessary for successful translation into the realm of clinical biodiagnostics. Moreover, regulatory approval procedures require stringent standardization of manufacturing processes to ensure consistency in aptamer synthesis, nanomaterial functionalization, and electrode preparation. Stability studies over long periods of time are also necessary to establish the shelf life, storage stability, and performance of these biosensors under different environmental conditions, especially since nanomaterials and surface-immobilized aptamers may degrade over time [70].

#### 3.1.6. Standardization and Manufacturing Scalability

Although the electrochemical nano-aptasensors developed in recent times are very robust, one of the disadvantages is in standardizing and reproducing the results in scaling up from the laboratory to industrial levels. Inconsistencies in the synthesis of nanomaterials, electrode fabrication, and aptamer functionalization are some of the disadvantages in scaling up aptasensors. Screen printing and roll-to-roll nanocoating are promising techniques for scaling up aptasensors, but standardizing the electrode surface, chemistry, and electrochemical properties is still a problem. Some of the techniques being developed to overcome this problem are automation, optimization of materials, and real-time monitoring considerations [70,71].

### 3.2. Knowledge Gaps

#### 3.2.1. Long-Term Stability and Shelf-Life Data

Currently, there is a lack of systematic investigations into the long-term storage stability of aptamer-functionalized electrodes at ambient conditions (temperature fluctuations, high humidity levels, exposure to dust, and the absence of cold chain conditions), which can have drastic effects on sensor performance. While short-term stability under controlled conditions in a laboratory setting is reported, there is a lack of investigations into long-term stability at room temperature or at higher temperatures. Degradation mechanisms of aptamer-functionalized electrodes in low-resource environments are not well understood. In order to bridge the gap between the laboratory environment and practical applications in low-resource environments, it is important to carry out comprehensive investigations into the stability of aptamer-functionalized electrodes [71].

#### 3.2.2. Mechanistic Understanding of Aptamer–Bacteria Interactions

Although SELEX-derived aptamers are known for their specificity and binding affinities towards their target molecules, the structural basis for their binding to whole bacterial cells, especially MDR strains like *S. aureus* or *P. aeruginosa*, still remains vaguely understood in detail from a molecular perspective. Most SELEX-based systems are based on the selection and screening of functional binders without a complete understanding of the precise three-dimensional conformational structures adopted by the aptamers during their binding process or the precise epitope targets on the bacterial surface. Without a complete understanding of the precise structural basis from a mechanistic perspective, such as through data from cryo-electron microscopy, NMR studies, or molecular dynamics simulations, optimization strategies for aptamers are still not well understood from a rational perspective and remain empirical in nature, which actively slows the pace of precise diagnostic and therapeutic strategies development for MDR bacteria [72].

#### 3.2.3. Standardization of Performance Metrics

Studies frequently report widely differing detection limits, measurement, sample matrices, and validation procedures, which makes it challenging to directly compare the analytical performance of electrochemical POC diagnostic platforms across laboratories and clinical contexts. Variations in electrode materials, signal amplification strategies, target biomarkers (e.g., nucleic acids, proteins, or whole cells), and sample preparation workflows further compound these inconsistencies, often leading to performance claims that are not evaluated under standardized or clinically relevant conditions. As a result, translating reported sensitivity or LOD values into meaningful clinical performance metrics (diagnostic accuracy in MDR pathogen detection) remains inconsistent. The absence of a universally accepted benchmarking framework, including standardized reference materials, agreed-upon reporting metrics, and harmonized validation protocols, limits objective cross-platform comparison and slows regulatory approval, clinical adoption, and large-scale implementation [73].

#### 3.2.4. Clinical Validation and Antimicrobial Susceptibility Testing (AST) Integration

Bacterial detection alone is not sufficient for effective analysis and clinical outcomes; AST remains a missing link that must complement bacterial detection to provide a complete and meaningful interpretation of the results. Although the electrochemical sensors have the ability to detect bacteria at a very fast rate, they still lack the ability to combine the results obtained from the detection of the bacteria with the results obtained from the AST. In the recent past, attempts have been made to combine the results obtained from the bacterial detection with the AST results. However, the attempts have been facing challenges in the miniaturization of the assays [74]. Moreover, rapid detection alone is insufficient for comprehensive AMR assessment, as pathogen identification alone does not necessarily reveal susceptibility or resistance mechanisms. The coupling of electrochemical sensing technologies with rapid phenotypic or genotypic AST has therefore become an important research goal, yet current implementations remain technically challenging. Limitations include difficulties in correlating electrochemical signal changes with functional resistance phenotypes, integrating complex biological assays into portable sensing devices, and achieving the sensitivity and specificity needed for clinical decision-making. As a result, the development of truly integrated systems that combine rapid detection with reliable resistance profiling is still in its early stages [75].

### 3.3. Considerations

#### 3.3.1. Design for Resource-Limited Settings

POC diagnostic systems are particularly useful in low-resource settings as they allow for rapid medical decision-making without the need for centralized laboratory facilities. To ensure widespread adoption, these systems must therefore focus on making affordability possible so that both patients and healthcare professionals can access testing technologies without incurring exorbitant costs. These systems should also require fewer sample preparation steps for rapid, direct testing of biological samples, which will not require extensive training. Battery-powered or low-energy operation is also critical for use in remote areas where access to stable electricity may not be available, and user-friendly interfaces will also help non-experts carry out tests correctly. Additionally, the use of disposable electrode strips and paper electrochemical platforms will greatly improve accessibility by reducing manufacturing costs, improving portability, and preventing cross-contamination, which will ultimately help ensure scalable deployment of reliable POC diagnostic testing [76].

#### 3.3.2. Surface Chemistry Optimization

Careful control of nanomaterial functionalization, aptamer orientation, and surface passivation is important to ensure minimal non-specific adsorption and maximized signal-to-noise ratio, as small changes can greatly influence performance. The precise functionalization of nanomaterial surfaces ensures that recognition molecules are uniformly and stably bound to the surface, maintaining their binding ability and preventing steric hindrance that could reduce target binding. Similarly, aptamer orientation is also important to prevent burial of active binding sites and increase target molecule accessibility. At the same time, effective surface passivation can prevent unwanted interactions from proteins, ions, or other environmental molecules that could otherwise contribute to background noise. By carefully controlling these factors, biosensing platforms can provide higher analytical sensitivity and accuracy, which is especially important in applications involving low-abundance targets or complex biological samples [77].

#### 3.3.3. Multiplexed and Syndromic Testing Approaches

It is envisioned that future diagnostic platforms should be able to simultaneously detect a variety of pathogens and AMR genes, which can significantly enhance the speed and accuracy of clinical decision-making, especially for complex diseases such as HAIs and sepsis. With the integration of advanced molecular sensing technologies, sample handling, and intelligent data analysis, future diagnostic platforms can offer healthcare professionals timely and comprehensive information regarding infections, which can significantly reduce the misuse of broad-spectrum antibiotics and expedite targeted therapy. This is particularly important in critical care environments where timely diagnosis is critical, as delayed diagnosis can result in rapid disease progression, increased mortality, and increased healthcare costs. Moreover, future diagnostic platforms must be able to address emerging microbial challenges, monitor resistance evolution, and be applicable for POC diagnostics to address diverse healthcare needs [78].

#### 3.3.4. Scalability, Sustainable Manufacturing, and Environmental Impact

The success of translation-based energy devices is contingent on the ability to reproducibly manufacture nanostructured electrodes on a large scale while ensuring consistent electrochemical properties across batches. However, scaling up from prototypes in the lab to actual production involves several aspects, such as nanoscale morphology control, aptamer direction, and nanomaterial-electrode interface consistency in large batches. Deviations in such aspects can be critical in terms of sensor reliability and performance. Roll-to-roll and automated fabrication are promising tools for the cost-effective production of the sensor. However, batch consistency and standardization in compliance with regulations are yet to be shown. Moreover, the inclusion of quality control measures and real-time POC monitoring considerations during the fabrication process would be necessary in order to take the research from the lab-scale to the actual production-scale [79].

Moreover, responsible deployment of advanced technologies requires attention to end-of-life environmental impacts, particularly through proper nanomaterial disposal, electronic waste management, and comprehensive lifecycle assessment. Nanomaterials can exhibit unique reactivity and persistence in ecosystems, making regulated handling and disposal essential to prevent soil and water contamination. Similarly, growing global reliance on electronic devices has intensified concerns about e-waste accumulation, which is why frameworks promoted by organizations encourage recycling, material recovery, and safe treatment of hazardous components. Integrating these practices into research, industry standards, and regulatory policies ensures that technological advancement proceeds in a manner aligned with long-term ecological protection and sustainable development goals (SDGs) [80].

## 4. Conclusive Remarks and Future Outlook

Electrochemical nano-aptasensors are no longer merely laboratory novelties: they have matured in sensitivity and target range and are projected to play a significant role in rapid MDR bacterial diagnostics under POC settings. Advances in aptamer design, nanomaterial signal amplification, microfluidics integration, and AI-assisted interpretation each play a part in overcoming technical challenges. There has been promising sensitivity and specificity exhibited by small pilot trials with blood, urine, or wound fluid samples versus reference standard PCR or culture [62]. Recently, various electrochemical aptasensors have been patented, and some of these are advancing towards practical applications in the detection of pathogens and biomolecules. The various patents for electrochemical aptasensors (including nano-aptasensors) for MDR bacterial detection over the years have been summarized in Table 1. This provides a view into the advancements in electrochemical POC technologies, synergistic integration of nanomaterials and aptamers, and emerging sensor fabrication strategies towards MDR bacterial detection.

However, multicentre validation and approvals are not yet prevalent. Other challenges notably include significant knowledge gaps and limitations in the field of aptamer–bacteria interactions, batch-to-batch reproducibility, cost of disposable sensors per unit, long-term stability of nanocomposites under varying storage conditions, the establishment of standardized benchmarking and validation procedures, and the integration of rapid detection with AST [62,63]. Moreover, final translational milestones (robust proof in diverse patient populations, scale-up fabrication of reproducible nanoarchitectures, cost reduction, and regulatory clearance) remain the main bottlenecks. Future studies may also focus on discovering new bacterial biomarkers and resistance factors that are recognizable by aptamers, such as virulence factors, efflux pump regulators, quorum-sensing molecules, and metabolite signatures. The inclusion of these features may also improve multiplexed sensing, personalized medicine, and early intervention. Computer algorithms and high-throughput screening of bacterial proteomes and secretomes may also lead to the discovery of new aptamer targets that have not yet been explored in electrochemical POC assays, advancing the scientific and clinical applications of MDR bacterial diagnostics.

The principal priorities to be considered for rapid clinical translation and efficient diagnostic workflow integration include the following:

- Uniform and analytical validation procedures: Standardizing analytical parameters (LOD, specificity within matrices designated), inter-lab reproducibility exercises, and relevant endpoints that guide therapy decisions. Recent reviews recommend harmonized standards to make regulatory processes more streamlined [7,8,74].
- Multiplex platforms for rapid, diversified diagnostics: Products should reliably detect pathogen identity, key resistance genes/proteins, and, wherever possible, provide rapid phenotypic susceptibility results [15,78].
- Manufacturing and cost engineering: The transition from nanofabrication in the bench to scalable technologies (screen-printing, roll-to-roll nanocoating) in a bid to minimize cost per test would improve reproducibility. Industry and multidisciplinary collaborations will be essential to achieve these aspects [40,79].
- Early clinician–regulator engagement: Collaborations with clinicians and regulators to develop validation standards and cohorts acceptable to regulators and funders could prove effective. Robust and rigorous testing and field trials in low-resource and decentralized settings are particularly important for equitable accessibility [63,70,80].

Looking forward, the field is poised to benefit from the convergence of next-generation SELEX methodologies, advanced nanomaterials, AI/ML-powered signal processing, and cost-effective manufacturing technologies. These combined developments could facilitate the deployment of highly sensitive, multiplexed, and real-time POC platforms capable of addressing emerging MDR threats globally, particularly in resource-limited settings. Additionally, if the translational challenges (standardized validation, scale-up of production, clinical trials, and cost reduction) can be addressed, nanotechnology-augmented electrochemical aptasensors can achieve the dream of rapid, near-patient MDR bacterial diagnosis and beyond. This would reduce time to analysis and targeted therapy, reduce inappropriate antibiotic exposure (broad-spectrum antibiotic prescription and overuse or misuse), and improve global surveillance, all concrete benefits that can aid in addressing the immediate major threat that AMR poses.

## Figures and Tables

**Table 1:** Overview of various patents in the field of electrochemical aptasensors (including nano-aptasensors) for MDR bacterial detection.

Patent Landscape (2007–2026)				
Patent Number	Year	Title	Description	Ref.
US20090087867A1	2007	Biosensor	The invention provides a biosensor comprising a microbe-binding aptamer(s) in the Substrate recognition element. It is possible to obtain a stabilized biosensor wherein the detection sensitivity for the target microbe (*Streptococcus mutans*) is not impaired, depending on the storage condition or measuring sample.	[81]
WO2010039941A9	2010	Bionanosensor detection device	The device comprises a bio-nanosensor element comprising ssDNA primed nanotubes, either single-walled or multi-walled. The method comprises contacting the bio-nanosensor element with a test solution potentially containing DNA of interest. DNA of interest that hybridizes to the ssDNA results in a measurable change in the electrical properties of the bio-nanosensor. Correlations between the results provided by the device and the presence of disease states can result in rapid diagnosis of diseases such as Lyme disease or foodborne infections such as salmonellosis.	[82]
WO2011133694A2	2011	Method and apparatus for forming an automated sampling device for the detection of Salmonella enterica utilizing an electrochemical aptamer biosensor	An aptamer-based solid-state electrochemical biosensor for label-free detection of *Salmonella enterica* serovars utilizing immobilized aptamers. The device is realized by forming a matrix array of parallel capacitors, thus allowing the realization of low-cost, portable, fully integrated devices. Protein-aptamer binding modulates the threshold voltage of a circuit, changing the impedance (capacitance) of the circuit.	[83]
WO2014166558A1	2014	DNA aptamers to diagnose *Mycobacterium tuberculosis* bacteria and treat tuberculosis disease, specific for *M. tuberculosis* bacteria	The present invention relates to the rapid diagnosis of *M. tuberculosis* and the treatment of tuberculosis using DNA aptamers.	[84]
WO2016112079A1	2016	Microfluidic aptasensor including a graphene nanosensor	Graphene nanosensors for monitoring a target analyte utilizing anti-target analyte aptamers can include a single conductance sensor on a substrate platform, where the graphene sensor can be functionalized with aptamers for binding the target analyte, or alternatively, a nanosensor can include microbeads functionalized with aptamers which can allow for selective enrichment and isocratic elution of the target analyte, where the concentration of the enriched target analyte can be measured on a graphene surface functionalized with a target analyte of interest.	[85]
US10309921B2	2019	Label-free electrochemical biosensor	The current invention pertains to electrochemical biosensors. The electrochemical biosensor of the current invention comprises the following: (a) A sensing electrode having attached to its surface a binding agent capable of specifically binding to the analyte to form a binding agent–analyte complex, and wherein the binding of the analyte to the binding agent alters the electron transfer properties at the sensing electrode surface, thereby providing a change in the electrochemical response at the sensing electrode surface proportional to the number of binding agent–analyte complexes; (b) A test equipment capable of measuring the electrochemical response at the sensing electrode surface. The binding agent can be a binding protein, an antibody, or an aptamer, and the analyte can be a biomolecule. Accordingly, the current invention provides a method of detecting the presence or assessing the likelihood of development of a disease associated with an abnormal level of a biomolecule in a subject.	[86]
WO2020100159A1	2020	A novel aptamer and an electrochemical biosensor for the rapid detection and diagnosis of tuberculous meningitis	The invention provides a novel HspX-specific aptamer, an electrochemical biosensor based on an HspX-specific aptamer for rapid and sensitive diagnosis of *Tuberculosis meningitis*, and methods of detecting Tuberculous meningitis.	[87]
US11782011B2	2021	Ultrasensitive electrochemical biosensors	An electrochemical biosensor includes a working electrode modified with a redox polymer and an amine-terminated capture aptamer specific for a particular detection target. The binding sequence of the capture aptamer is also complementary to part of a second ssDNA, which is labeled with HRP (horseradish peroxidase). The capture aptamer will form dsDNA with the HRP-labeled ssDNA and bring HRP into electrical contact with the redox polymer and the electrode.	[88]
EP4365292A1	2022	Aptamer for the detection of the microorganism *Bacillus subtilis*	The present invention relates to an aptamer, i.e., a single-stranded DNA or RNA sequence, for the detection of the microorganism *Bacillus subtilis.*	[89]
WO2023137010A3	2023	Electrochemical aptamer sensors with signal amplification via multiple redox tags	A device for detecting the presence of, or measuring the concentration or amount of, at least one analyte in a sample fluid is disclosed. The device includes at least one electrode, a sample fluid, and a plurality of affinity-based probes capable of binding to the analyte, wherein the affinity-based probes each carry a plurality of redox tags.	[90]
WO2025122621A1	2024	Compositions and methods related to aptamers and aptamer-based sensors	The present disclosure provides compositions and methods related to aptamers and aptamer-based sensors.	[91]
TR2025011523A1	2025	Method for developing nanofiber-based biosensors for the detection of pathogenic microorganisms	The invention relates to a biosensor system and production method for the rapid and sensitive detection of pathogenic microorganisms, which can be used in areas such as food safety, environmental monitoring, and medical diagnosis.	[92]
US12517126B2	2026	Systems and methods for detecting a pathogenic organism	A method of detecting the presence, amount, and/or type of a pathogenic organism in a substrate is provided. The method is effected by contacting a sample suspected of containing the pathogenic organism or a portion thereof with an electrode, thereafter contacting the electrode with an aptamer that selectively binds to said pathogenic organism; thereafter contacting the electrode with an agent that participates in an electrochemically detectable reaction, and thereafter performing the electrochemical reaction while using the electrode.	[93]
US20260009789A1	2026	Methods and devices for detecting a pathogen and its molecular components	An example system for improving detection of a pathogen includes a biosensor device comprising a detection chip and at least one probe that specifically recognizes a pathogen, where the detection chip comprises a graphene field-effect transistor (FET) chip and the probe, which comprises an aptamer, specifically binds to a DNA, RNA, or protein associated with the pathogen.	[94]

## Data Availability

No new data were created or analyzed in this study. Data sharing is not applicable to this article.

## Abbreviations

The following abbreviations are used in this manuscript:

AI	Artificial Intelligence
AMR	Antimicrobial Resistance
AuNPs	Gold Nanoparticles
CDC	Centers for Disease Control and Prevention
CNTs	Carbon Nanotubes
EBs	Electrochemical Biosensors
EHRs	Electronic Health Records
GCE	Glassy Carbon Electrode
GFETs	Graphene-Based Field-Effect Transistors
HAIs	Healthcare-Associated Infections
ICUs	Intensive Care Units
IoMT	Internet of Medical Things
IoT	Internet of Things
LODs	Limits of Detection
LPS	Lipopolysaccharides
MB	Methylene Blue
MDR	Multidrug-Resistant
MEMS	Microelectromechanical Systems
ML	Machine Learning
MOFs	Metal–Organic Frameworks
MRSA	Methicillin-resistant *Staphylococcus aureus*
MWCNTs	Multi-Walled Carbon Nanotubes
POC	Point-of-Care
SDGs	Sustainable Development Goals
SELEX	Systematic Evolution of Ligands by Exponential Enrichment
SPCE	Screen-Printed Carbon Electrode
SPR	Surface Plasmon Resonance
Td	DNA Tetrahedron
WHO	World Health Organization
